# Recommendations from the European Working Group for Value Assessment and Funding Processes in Rare Diseases (ORPH-VAL)

**DOI:** 10.1186/s13023-017-0601-9

**Published:** 2017-03-10

**Authors:** Lieven Annemans, Ségolène Aymé, Yann Le Cam, Karen Facey, Penilla Gunther, Elena Nicod, Michele Reni, Jean-Louis Roux, Michael Schlander, David Taylor, Carlo Tomino, Josep Torrent-Farnell, Sheela Upadhyaya, Adam Hutchings, Lugdivine Le Dez

**Affiliations:** 1 Department of Public Health, Ghent University, Ghent, Belgium; 2 ICM, CNRS UMR 7225––Inserm U 1127––UPMC-P6 UMR S 1127, Paris, France; 3 EURORDIS-Rare Diseases Europe, Paris, France; 4 University of Edinburgh, Edinburgh, Scotland; 5 Swedish Parliament, Stockholm, Sweden; 6 Centre for Research on Health and Social Care Management (CERGAS), Bocconi University, Milan, Italy; 7 IRCCS Ospedale San Raffaele Scientific Institute, Milan, Italy; 8 EURORDIS-Rare Diseases Europe, Paris, France; 9 Health Economics at the University of Heidelberg, Heidelberg, Germany; 10 Division of Health Economics at the German Cancer Research Center (DKFZ), Heidelberg, Germany; 11 Institute for Innovation & Valuation in Health Care, Wiesbaden, Germany; 12 Pharmaceutical and Public Health Policy, University College London (UCL), London, UK; 13 Clinical Research at IRCSS San Raffaele, Rome, Italy; 14 Clinical Pharmacology and Therapeutics, Autonomous University of Barcelona, Barcelona, Spain; 15 NICE, Manchester, UK; 16 Dolon Ltd, London, UK; 17 European government relations and public policy at Celgene, Brussels, Belgium

**Keywords:** Orphan medicinal products, Rare diseases, Pricing and reimbursement, Health technology assessment, Value assessment, Guidelines

## Abstract

Rare diseases are an important public health issue with high unmet need. The introduction of the EU Regulation on orphan medicinal products (OMP) has been successful in stimulating investment in the research and development of OMPs. Despite this advancement, patients do not have universal access to these new medicines. There are many factors that affect OMP uptake, but one of the most important is the difficulty of making pricing and reimbursement (P&R) decisions in rare diseases. Until now, there has been little consensus on the most appropriate assessment criteria, perspective or appraisal process. This paper proposes nine principles to help improve the consistency of OMP P&R assessment in Europe and ensure that value assessment, pricing and funding processes reflect the specificities of rare diseases and contribute to both the sustainability of healthcare systems and the sustainability of innovation in this field. These recommendations are the output of the European Working Group for Value Assessment and Funding Processes in Rare Diseases (ORPH-VAL), a collaboration between rare disease experts, patient representatives, academics, health technology﻿ assessment ﻿(HTA) practitioners, politicians and industry representatives. ORPH-VAL reached its recommendations through careful consideration of existing OMP P&R literature and through a wide consultation with expert stakeholders, including payers, regulators and patients. The principles cover four areas: OMP decision criteria, OMP decision process, OMP sustainable funding systems and European co-ordination. This paper also presents a guide to the core elements of value relevant to OMPs that should be consistently considered in all OMP appraisals. The principles outlined in this paper may be helpful in drawing together an emerging consensus on this topic and identifying areas where consistency in payer approach could be achievable and beneficial. All stakeholders have an obligation to work together to ensure that the promise of OMP’s is realised.

**Electronic supplementary material:**

The online version of this article (doi:10.1186/s13023-017-0601-9) contains supplementary material, which is available to authorized users.

## Background

The European Union (EU) regulation on Orphan Medicinal Products (OMPs) EC 141/2000 has been vital for stimulating investment into OMP research and development (R&D), furthering an EU-wide aim of ensuring that patients with rare diseases have equity of access to effective treatment [1, 2]. To date, the European Medicines Agency (EMA) has authorised 129 OMPs and 1,435 as yet unapproved products have OMP designation [3]. Rare diseases are an important public health issue and it is important that patients with these diseases have access to safe and effective therapies in the same way as others suffering from more common diseases [4]. Once regulatory authorisation has been granted to an OMP by the EMA, it is essential for patients to benefit from it within the shortest timeline and this should be consistent across Europe. Whether this goal is achieved is influenced by the pricing of these therapies and by the mechanisms used by national healthcare decision-makers to assess, reimburse and fund them [1, 2, 4–7]. A 2009 study found that, of 43 EMA-approved OMPs, the proportion reimbursed varied from 56 to 91% across seven Western-European countries [1, 8]. Another evaluation of 10 European countries observed a difference in reimbursement between countries according to income level, with France, Netherlands and Denmark reimbursing 90% of 60 authorised OMPs, while Spain, Greece and Romania reimbursed only one-third [9].

P&R processes for OMPs are particularly challenging due to the inherent characteristics of rare diseases and the scarcity of knowledge and expertise on the natural history of these conditions [10, 11]. Clinical trial development is also complicated due to small and typically heterogeneous patient populations, resulting in difficulties in patient identification and recruitment, and lack of surrogate end-points to predict longer term outcomes [12]. Therefore health technology assessment (HTA) agencies often have to assess OMP value – the benefit from treatment for patients, healthcare systems and society – in the presence of evidential uncertainty [10, 11].

The small size of the target patient population – OMP regulation in Europe stipulates a prevalence of less than 5 in 10,000 [13] – means that the per-patient price of OMPs is higher than for medicines for more common conditions [14]. As a consequence, OMPs assessed in systems that use cost-effectiveness analysis have often failed to meet accepted thresholds, particularly where the prevalence is at the lower end of the spectrum of rare diseases [11, 15]. Uncertainty about the true size of rare disease patient populations creates financial risk for payers and compounds broader concerns about the ongoing cost of funding the growing number of treatments for rare diseases [5, 16, 17].

Some HTA bodies and payers have reacted to these challenges by creating supplementary OMP-specific assessment mechanisms (e.g. NICE Highly Specialised Technology Programme in England and the Life Savings Drugs Program in Australia) or by adapting existing P&R processes (e.g. OMP special status in benefit assessments in Germany and the PACE process at the Scottish Medicines Consortium) [18, 19]. More often, decisions regarding OMPs are made following the same process as for other medicines.

The diversity of P&R pathways for OMPs across European countries can represent a challenge to all manufacturers of medicines, and this is particularly true for OMPs. While there is some consistency in the core clinical information considered by HTA agencies, there are considerable disparities in how data is interpreted, the use of economic analysis, the perspective of the evaluation and the extent to which patients and healthcare professionals (HCPs) are involved, amongst other factors [11, 18, 20, 21].

These differences in assessment methodology partly reflect a lack of consensus amongst policy makers on the optimal P&R framework for OMPs and how to overcome the challenges that rare diseases represent to HTA agencies. Many European countries are reviewing P&R processes for OMPs, providing an opportunity to seek closer alignment on the fundamental aspects of such systems [22, 23].

In this paper we propose nine principles to help improve the consistency of OMP P&R in Europe and ensure that it reflects the inherent characteristics of rare diseases. The principles cover issues relating to value assessment, P&R, and funding processes for OMPs, grouping them in four parts: decision criteria, decision processes, sustainable funding and European co-ordination.

Moreover, to help improve consistency in the decision criteria used to assess the value of OMPs between countries, we propose a set of core elements that together constitute the value of an OMP. This paper does not represent an attempt to define a single P&R framework for European countries, but rather to propose underlying principles that should be common to all, regardless of the mechanism used in each country to make decisions.

**Fig. 1:**
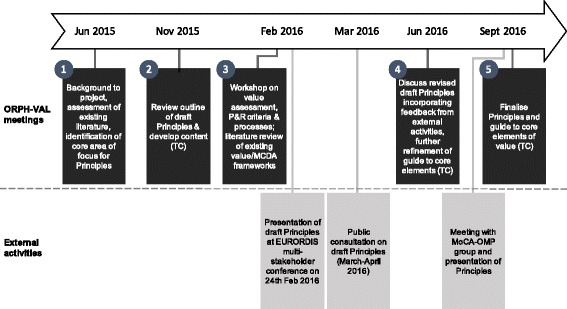
Process through which the Principles were developed. MoCA-OMP: mechanism of coordinated access to orphan medicinal products; MCDA: multi-criteria decision analysis; P&R: Pricing and reimbursement; TC: teleconference

The principles described herein are recommendations from the 15 authors of this article, who are designated as the European Working Group for Value Assessment and Funding Processes in Rare Diseases (ORPH-VAL). This Working Group is a collaboration between rare disease experts across seven European countries, including HTA practitioners, physicians, patient representatives, academics, politicians and industry representatives. A detailed description of each Working Group member’s experience relevant to this topic is provided in the declaration section. The experts were selected to achieve a breadth of geographical and disciplinary perspective and based on their active involvement in the field of rare diseases and willingness to participate in the initiative on a voluntary basis. ORPH-VAL reached its recommended principles through an iterative process of assessment of existing OMP guidelines and frameworks, identification of core themes, formulation of draft principles, internal and external review, and subsequent refinement. The Working Group members participated in five workshops (two face to face, three via teleconference) between June 2015 and September 2016 (Fig. 1). Wherever possible, ORPH-VAL has built upon, or aligned with, outputs from existing initiatives in this area, such as the Mechanisms of Co-ordinated Access to Orphan Medicinal Products (MoCA-OMP) and EUnetHTA Joint Action 2.

The core elements of OMP value were selected from a systematic review of proposed frameworks for rare diseases and non-rare diseases [24]. Table 1 shows the frameworks reviewed by ORPH-VAL.

**Table 1:** Overview of value assessment frameworks relevant to OMPs

	Name of framework	Year of publication	Country or region	Description of framework	Number of domains and/or criteria in framework^a^	Reference
OMP-specific frameworks	Transparent Value Framework (TVF) by the Working Group on Mechanism of Coordinated Access to Orphan Medicinal. Products (MoCA-OMP)	2012	Europe	Instrument to assist value-based pricing in a transparent way listing important criteria contributing to the value of an OMP. Semi-quantitative framework for determining the degree to which the individual criteria are met	4 criteria	[25]
	Framework by Hughes-Wilson et al.	2012	Europe	Assessment system based on several weighted evaluation criteria and their corresponding potential parameters, which would serve as a tool for Member States, to value an OMP	9 criteria	[26]
	Multi-criteria decision analysis (MCDA) Framework by Sussex et al.	2013	Europe	Framework for informing value definition of OMPs and providing an explicit understanding of trade-offs for decisions on their eligibility for funding	2 domains 8 criteria	[21]
	MCDA for ranking rare diseases in Russia by Fedyaeva et al.	2014	Russia	MCDA used to estimate relative importance of 16 criteria to rank and prioritise rare diseases	2 domains 16 criteria	[27]
	MCDA framework by Schey et al.	2014	Europe	MCDA framework using criteria suggested by Hughes-Wilson et al. A supplementary literature review identified other attributes described in the application of MCDA in rare diseases.	13 criteria	[28]
	Decision-making framework by Paulden et al.	2015	Worldwide	Decision factors related to the reimbursement of OMPs were identified in a scoping review and synthesised into a decision-making framework	3 domains 29 criteria	[29]
	EVIDEM (EVIdence based Decision-Making) framework by Wagner et al.	2015	Worldwide	Quantitative MCDA framework to address rare disease issues and policies The framework is regularly updated, the latest version is V3.1	6 domains 15 criteria	[30]
Non-OMP frameworks	Conceptual MCDA framework by Kanavos et al.	2013	N/A	MCDA framework to assess the value of a new drug in a value-based context	4 domains 12 criteria	[31]
	MCDA framework in Hungary by Endrei et al.	2014	Hungary	MCDA framework used in the evaluation of medical technologies in hospitals	6 criteria	[32]
	Framework by Williams et al.	2014	Case studies from the UK, Germany, Spain to illustrate the MCDA process	Process for using MCDA by a pharmaceutical company to estimate the probability of a positive recommendation for reimbursement for a new drug given drug and environmental attributes	Participants were asked to select up to 10 criteria out of a long list of reimbursement criteria	[33]
	MCDA framework in Germany by Wahlster et al.	2015	Germany	Case study exploring the use of an MCDA approach to appraise a pulmonary heart sensor in Germany using the EVIDEM V2.2 framework	Not mentioned (EVIDEM framework V2.2)	[34]
	The European Society for Medical Oncology magnitude of Clinical Benefit Scale (ESMO-MCBS)	2015	Europe	Tool to assess the magnitude of clinical benefit for cancer medicines to derive a relative ranking between new treatments	3 to 5 criteria Number of criteria depend on the type of therapy (curative vs palliative)	[35]
	The American Society of Clinical Oncology (ASCO) Conceptual Framework	2015	USA	Framework for assessing the value of treatment options and was designed to eventually assist in facilitating shared decision making with patients about clinical benefits.	3 criteria	[36]

^a^In given frameworks, individual value criteria have been grouped into domains/broad clusters

Elements of value proposed in the literature were presented to the Working Group according to the frequency of occurrence and grouped by theme (e.g. disease-related, clinical, economic) and by perspective (patient, healthcare system, society). From this starting point, ORPH-VAL members were split into two groups which separately prioritised the core elements, before seeking consensus between the groups. Two sets of elements were considered: 1) elements that inform on the intrinsic value of an OMP and as such should be accounted for by decision making committees during the deliberative process; 2) elements that are beyond product value but are likely to inform or influence the P&R decision.

**Fig. 2:**
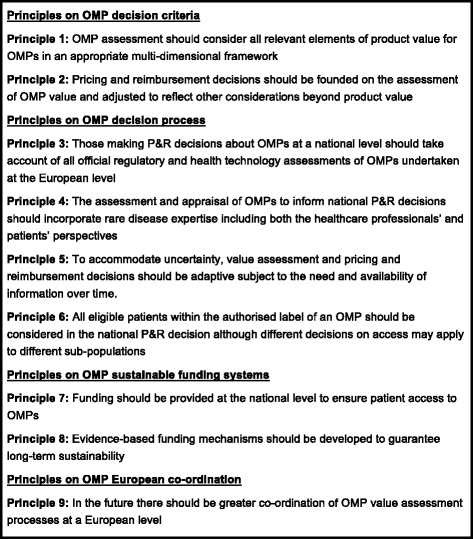
Summary of Principles for value assessment and funding processes in rare diseases

ORPH-VAL refined its principles through feedback from other European rare disease experts and stakeholders (Fig. 1). A draft version of the principles was presented and debated during the EURORDIS Multi-Stakeholder Symposium on the 24th of February 2016. A public consultation of the draft principles was organised through EURORDIS and publicised through OrphaNews on the 22nd March 2016. Interested participants were invited to complete an online survey and provide their comments on the document. Finally, ORPH-VAL had an opportunity to present and discuss the principles with the MoCA-OMP Group in September 2016. Feedback from all commentators was collated into themes for consideration by ORPH-VAL before being incorporated into a second draft of the principles. The nine principles are summarised in Fig. 2.

## Principles on OMP decision criteria

**Table:** 

**Fig. 3:**
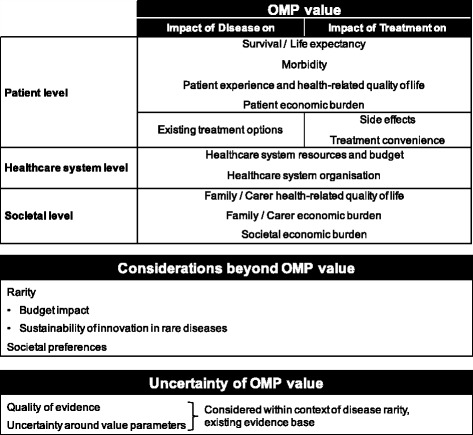
Guide to core elements of value relevant to pricing and reimbursement decisions in rare diseases. See Additional file 1 for glossary of terms on the core elements of value

**PRINCIPLE 1:** **OMP assessment should consider all relevant elements of product value in an appropriate multi-dimensional framework**	
a) Decision-makers should consider OMP value from the perspective of patients, the healthcare system and wider society b) While the choice of value elements that are used to assess OMPs should be country specific, ORPH-VAL has proposed a set of core elements that should be common to all health systems (Fig. 3) c) HTA agencies and payers should make explicit which elements of value they prioritise, how the rarity of a disease influences their assessment, and how societal preferences are incorporated into their decisions	

Rare diseases have particular characteristics beyond the small number of patients affected. They often impact patients from birth, affect multiple organ systems, are severely disabling, can greatly reduce life expectancy and impair physical and mental abilities [37, 38]. These impairments affect health-related quality of life (HRQoL) and can impact an individual's ability to benefit from education and secure employment [39]. In a survey of 2,500 patients with chronic diseases, patients with rare disorders had the worst experience in terms of loss of social and economic opportunities and of access to medical care [39].

Rare diseases also often have a disproportionate impact beyond the patient. Many rare diseases are genetic, meaning more than one member of a family can be affected [39]. Rare diseases also pose a considerable burden on caregivers [37, 39]. The severity of rare diseases means patients often require intensive care, usually from a family member, which reduces their ability to work. The lack of specialist support and information on the disease increases the burden for patients and their families. At a wider level, rare diseases require substantial healthcare and social care system resources [38, 40–42]. Given the multi-dimensional nature of the burden of rare diseases and the wide range of stakeholders affected, it is important that decision makers assess the value of OMPs holistically, including the perspectives of patients, the healthcare system and wider society (including family and carers).

The elements that are considered within P&R decisions vary across European countries [43]. ORPH-VAL has proposed a set of core elements it believes should be common to all systems (Fig. 3; a detailed description of the elements and explanation of the terms used is included in Additional file 1). The elements described in Fig. 3 represent proposed domains of value. ORPH-VAL did not seek to define metrics for how these elements should be measured, nor value thresholds or weights indicating the relative importance of different elements. Such factors, which relate to the creation of societal value, should be informed by, and aligned with, societal preferences of the population in the respective country. While information on potential international variation in societal value judgments is still limited, there is a growing body of literature documenting the relevance and breadth of societal preferences [44, 45].

With a recognition of the importance of transparency in the evaluation process, P&R frameworks should be clear about the elements of value that are considered within the value assessment process and their relative importance in determining the overall decision [46, 47]. It should be explicit how these decisions are informed so as to ensure legitimacy and to provide clear guidance to OMP developers about the types of products that society wishes them to develop [48].

Multi-criteria decision analytic (MCDA) frameworks have been proposed for rare diseases [21, 25, 26, 29, 30]. These frameworks have the advantages of providing sufficient flexibility to incorporate all value elements of importance and, as shown in a pilot study of a rare disease MCDA framework, are capable of incorporating different stakeholder perspectives [21]. In contrast, where cost-effectiveness frameworks have been used to assess OMPs, they have often failed to be sufficiently flexible to capture all relevant elements and perspectives [49–51].

**Table:** 

**PRINCIPLE 2: Pricing and reimbursement decisions should be founded on the assessment of OMP value and adjusted to reflect other considerations beyond product value**	
a) Reimbursement decisions should be based on the product value delivered by an OMP as described in Principle 1 b) The price should, among other elements, be informed by considering the magnitude of the product value in light of price-value precedents for other specialist technologies and medicines c) P&R decisions should reflect the value that the EU attributes to OMPs through the incentives put in place to develop them d) Beyond OMP value, P&R status should be modulated to reflect other considerations, such as societal preferences, rarity, budget impact and sustainability of innovation in rare diseases e) In countries where cost-effectiveness is applied to assess value for money, the incremental cost-effectiveness ratio (ICER) thresholds should be modulated to reflect: • all specificities of rare diseases (including rarity, unmet therapeutic need and societal preferences) • the need to maintain innovation in rare diseases f) P&R decisions should aim at contributing to the right balance between allowing sufficient revenue generation to incentivise new research investment in rare diseases and attract private funders, while maximising value for money for healthcare systems	

The OMP value assessment process, as described in Principle 1, should form the basis of P&R decisions. These two components – value assessment and P&R appraisal – are often undertaken separately and consecutively in many European systems, such as in France and Germany [52, 53]. In order to translate an assessment of value into a P&R decision, it is necessary to interpret the added value generated by a new OMP relative to an appropriate price benchmark. The P&R decision should therefore consider the value for money of a new OMP in light of price precedents for other specialist medicines that offer comparable value. These comparator products should share similar fundamental characteristics to the OMP under evaluation in respect to disease severity, number of patients and product attributes [5].

Beyond the value of the OMP, other considerations are relevant to P&R decisions, including the size of the expected budget impact (across all approved indications, if more than one) and societal preferences concerning the value of new treatments for a given rare disease [30, 47, 54–56].

The size of the patient population for an authorised treatment is an important consideration in P&R decision making. Rarer diseases are associated with greater evidential uncertainty compared with more common diseases as there is less information on the natural history of disease and smaller patient numbers in clinical trials [57]. Rarity is therefore pertinent to the consideration of the uncertainty around the value estimate. The size of the patient population is also a major determinant of the budget impact for payers and the revenue potential for manufacturers and thus is directly relevant to the P&R decision [7, 58]. OMP legislation was an explicit recognition that incentives to invest in developing treatments for rare diseases are lower than for more common diseases (a smaller patient population results in a lower potential return on investment) [2, 59]. OMP pricing has subsequently been observed to be correlated with disease prevalence, with higher per patient prices in the rarest diseases [60]. Studies of societal preferences for funding treatments for rare diseases have not shown clear support for prioritising rarity alone in P&R decisions, however there is a preference for reducing inequalities in health outcomes [61–63]. Given that the degree of rarity directly affects the financial incentive to develop treatments in a given disease area, it is only possible to address health inequalities in small populations if rarity is taken into account.

Cost effectiveness analysis (particularly when restricted to a health service perspective) is not optimal for the assessment of OMPs, as the QALY fails to account for all of the core elements of value relevant to rare diseases [49–51]. If it is to be used to inform assessments of OMPs, the ICER thresholds should be modulated to reflect all the specificities of rare diseases as described in Fig. 3. Countries that use strict and non-modulated ICER thresholds to guide reimbursement decisions have a poor record of approving OMPs (e.g. Canada) [49]. For OMPs to be approved in systems using ICERs for decision making, payers will need to adopt variable thresholds at levels higher than that applied historically [14, 20].

In general, P&R decisions should balance the need to enable revenue generation to incentivise new research investment in rare diseases with the need to maximise value for money for healthcare systems. But OMPs prices should be such that all products that provide clinically meaningful added benefits (i.e. that are approved by the Committee for Orphan Medicinal Products [COMP]/EMA) are commercially feasible and incentivise continued investment in rare diseases. At the same time, the P&R decision must also consider the affordability of the expenditure and be aligned with the sustainability of healthcare system finances.

## Principles on OMP decision process

**Table:** 

**PRINCIPLE 3: Those making P&R decisions about OMPs at a national level should take account of all official regulatory and health technology assessments of OMPs undertaken at the European level**	
a) National P&R agencies should build on the decisions and recommendations at a European level, including: • The Committee for Orphan Medicinal Products (COMP)’s assessment of significant benefit and prevalence • The EMA’s European Public Assessment Report and Summary of Product Characteristics • Relative effectiveness assessments undertaken by the European network for HTA	

To ensure consistency and alignment between Member States, avoid duplication of evaluations, and improve resource utilisation, P&R agencies should build on the official decisions and recommendations made for OMPs at a European level, tailoring and adapting these assessments to their local environment. Currently, the duplication of regulatory and HTA activities at a national level contributes to the lengthy and often variable access to OMPs across Member States [64].

Official decisions and recommendations include the COMP’s assessment of significant benefit and prevalence, the EMA’s European Public Assessment Report (EPAR) and the relative effectiveness assessments undertaken by the European network on health technology assessment (EUnetHTA). Payers should also account for early conditional and exceptional marketing authorisations as well as the early scientific advice provided by the EMA. European legislation recommends Member States use COMP/EMA assessments to guide and align national decision making, helping build on existing, central regulatory European assessments.

This approach offers several benefits. European-level health authorities have the greatest concentration of rare-disease expertise and, importantly, COMP offers specific rare disease guidance (conversely, national level assessment authorities are typically more generalised). This also ensures Member States can utilise clinical experts from the specialist centres across Europe [2, 4, 65]. Avoiding duplication of effort and reducing existing asymmetries in assessments may improve resource utilisation and, subsequently, reduce the time to OMP decision-making.

While there is a need to ensure national assessments and decisions reflect the local population’s needs, there is a strong argument that, in the case of rare diseases, the centralised knowledge, data and expertise that is pooled at the European level leads to stronger decision processes than those restricted to information available locally. Where central and national opinions differ, with contradictory conclusions, there is a need to acknowledge the importance of this and find consistency between assessments.

**Table:** 

**PRINCIPLE 4: The assessment and appraisal of OMPs to inform national P&R decisions should incorporate rare disease expertise including both the healthcare professionals’ (HCP) and patients’ perspectives**	
a) HCPs and patients and their carers should be involved in the value assessment in the following ways: • Disease-specific expert physicians (and other relevant academic specialists) should be involved from scoping of an assessment through to appraisal determination by the bodies that assess and appraise OMP • Systematic representation of patient associations in meetings that assess and appraise OMPs • Disease-specific patient representatives should be involved throughout the process and given appropriate training and support to contribute fully	

HCPs, patients (and their carers) should be involved in the value assessment (HTA) and appraisal (P&R decision making) of OMPs, offering an important insight into the real-world experience of a rare disease [5, 29, 66–71]. The nature of rare diseases mean that the involvement of patient and physician experts is even more important than in assessments of treatments for non-rare diseases. There is often limited information on the natural history and burden of a rare disease, and current standard of care treatments may be poorly established with little evidence of effectiveness [72, 73]. These stakeholders can help authorities understand what outcomes are relevant in a disease and what level of improvement is clinically meaningful. HCPs and patients have the expertise and experience to discuss HRQoL, burden of disease and patient preferences [67, 74, 75]. Clinical experts and patients may also help interpret the relevance of trial data, where endpoints might be unusual or not validated in the disease in question. They can help assessment authorities understand why surrogate endpoints have been used and what they mean [76, 77].

In addition to trial design interpretation, clinical experts and patient groups can help inform estimates of prevalence and potential patient uptake on treatment. Patient associations have previously provided very accurate estimates of patients (e.g. the MPS Society for elosulfase alfa in the UK [78]). Experts may also help to establish the natural history of the rare disease, its symptomology and burden, in the absence of observational data. For example, patient interviews and surveys have been instrumental in establishing knowledge about the ways in which rare diseases affect patients, their families and the costs they impose on wider society [79]. Finally, patients and HCPs can provide insights into the current patient treatment pathways and identify issues with current treatments and expectations from new therapies [69, 75, 77].

The involvement of HCPs and patients in value assessment can be implemented in a variety of ways. For example, disease-specific expert physicians (and other relevant academic specialists) can input directly into the assessment and appraisal process. Patients can be systematically represented in bodies that assess and appraise OMPs as part of an overarching patient association. Disease-specific patient representatives could be involved throughout the process, provided that they have received appropriate training and support to contribute fully. EUPATI (the European Patient’s Academy) provides training courses for patients with the aim of enhancing their skills and enabling them to contribute to medicines research and development. Such initiatives should be supported and supplemented with process-specific training at national levels [80].

To this end, clear, consistent guidelines should be developed, enabling stakeholders (in particular patient associations), to prepare submissions and be in a position to respond to the appraisal process. For example, generic patient group submission templates, such as those developed by Health Technology Assessment International (HTAi), can help patient groups identify the type of information that HTA agencies require in regard to patient and caregiver experiences, living with the disease, its management and unmet needs [81].

**Table:** 

**PRINCIPLE 5: To accommodate uncertainty, value assessment and pricing and reimbursement decisions should be adaptive subject to the need and availability of information over time**	
a) Given the nature of rare diseases, there is inherent uncertainty around all elements of product value. When assessing value, payers should consider this uncertainty in light of: • disease prevalence • disease severity and unmet need • amount of prior research conducted in the disease • extent to which the manufacturer has taken reasonable steps to minimise uncertainty b) To account for clinical and economic uncertainty, value assessment processes need to be adaptive (i.e. contingent), where necessary, and continuous rather than binary at the point of launch c) Pricing and reimbursement decisions should allow movement both up and down with newly generated evidence on value d) Where adaptive processes are required, all parties (payers, HTA agencies, involved HCPs, patients and industry) need to agree on this iterative process and clearly document the following: • the evidence required and milestones for each step of the assessment • the implications of not meeting the requirements and expectations initially agreed • each stakeholder’s shared responsibility to collect and evaluate the data e) Where possible, the collection and analysis of real-world data should be co-ordinated at a European or international level and should be integrated in disease level registries and databases: • to obtain more European consistency in the continuous assessment and appraisal of OMPs • to collect data on the true prevalence of a given rare disease in order to minimise financial uncertainty for payers	

Given the nature of rare diseases, uncertainty is inherent in all elements of product value. When assessing value, payers should consider this uncertainty in light of the disease prevalence and the level of existing knowledge and evidence about the disease. Typically, rare diseases with the lowest prevalence are associated with greater uncertainty compared with rare diseases with a higher prevalence [20].

At the time of launch, it should be recognised that, due to the size of the patient population and limited natural history data, there will often be incomplete information to provide certainty around the long-term clinical benefit and overall value of a new OMP [57]. In those cases there will be a need for a continuum of evidence generation. Further data collection will often be required post-launch to enhance the understanding of the clinical benefit and the overall value and to reduce uncertainty. Systematically collecting data from registries as well as implementing managed access schemes (where possible) could help mitigate the uncertainties and fill data gaps. This need for incremental evidence generation has been recognised through conditional approval regulatory processes [82].

Value assessment processes need to be adaptive (i.e. contingent) where necessary and continuous, rather than binary at the point of launch. An adaptive value assessment process would take into account the nature of the R&D challenge in rare diseases. Furthermore, it would contribute to better access to treatments for patients and would allow the building of evidence in clinical practice. P&R decisions may also be adaptive (i.e. increased or decreased) based on the data provided. While there are precedents for adaptive P&R mechanisms in Europe, there is some scepticism about their effectiveness historically (from both payers and industry) and a need for more robust design and implementation in future [10, 83].

Adaptive pathways to OMP assessment have been increasingly mentioned in recent policy debates and have been piloted at the European level [84]. Where adaptive processes are required, a framework for shared agreement between stakeholders should be developed to identify key responsibilities of all parties. Data collected should be independently validated and reviewed to maintain objectivity.

There has been a trend towards payers mandating country-specific registries (e.g. Germany, UK, France) to support conditional reimbursement decisions for OMPs with different data variables collected [64]. Where possible, the collection and analysis of real-world data should be co-ordinated at a European level and should be integrated in disease level registries and databases to obtain more consistency in the continuous assessment and appraisal of OMPs. This requires common definitions of outcomes to be collected as well as uniform sets of standards to guarantee data quality and completeness. It is essential to ensure that the duties and rights of every party involved in real-world data collection are respected. Therefore, financial responsibilities for collecting registry data should be shared proportionally between interested stakeholders as per the EUCERD recommendation on rare disease patient registration [85]. There should be the option to supplement this data with additional information that may be specific to Member States. EUnetHTA has notably put in place a common core protocol for Additional Evidence Generation (AEG) to set out the methodological basis for European cooperation in this field [86].

Estimating the likely budget impact of OMPs is a key challenge for payers [47, 55]. The greatest uncertainty in estimating budget impact concerns the number of patients who will receive treatment. This uncertainty stems from the challenges of estimating prevalence as a result of the poor diagnosis of rare disease patients and the lack of expertise in these diseases. Local prevalence data is often inadequate and there is an opportunity to reduce uncertainty through the pooling of epidemiological data at the European level. However, coherent systems are necessary to facilitate this collaboration between Member States and to allow for the co-ordination of post-launch data collection.

In advance of product launch, manufacturers, payers, HTA agencies, centres of expertise and European reference networks should collaborate to collect data on the true prevalence of a given rare disease in order to minimise financial uncertainty for payers. Such early collaboration will also ensure development of adequate capabilities for large scale post-launch data collection.

**Table:** 

**Principles 6: All eligible patients within the authorised label of an OMP should be considered in the national P&R decision although different decisions on access may apply to different sub-populations**	
a) Wherever possible, reimbursement decisions should seek to ensure that all patients specified in the product marketing authorisation should receive access to treatment b) Reimbursement may be reflective of situations where there is a broad spectrum of disease and clearly defined patient subgroups in which OMP value substantially differs	

The heterogeneous nature of rare diseases and small patient populations make it difficult to collect extensive data [57]. For ethical reasons, it may not be possible to generate data from certain subgroups of populations (e.g. children) who are often affected by rare diseases, but may not be allowed to participate in clinical trials [87]. Wherever possible, reimbursement decisions should seek to ensure that all patients specified in the product marketing authorisation should receive access to treatment. This should take into consideration how the uncertainty for a proportion of the population can be better addressed. This may be in the form of an adaptive pathway or managed access program.

Data usually does not allow for sub-grouping in a way that is statistically or clinically credible. However, where there is a broad spectrum of disease, and the financial impact of treatment is very high, it is reasonable to consider this in P&R decisions across patient subgroups.

## Principles on OMP sustainable funding systems

**Table:** 

**PRINCIPLE 7:** **Funding should be provided at the national level to ensure patient access to OMPs**	
a) Funding for OMPs should be co-ordinated at a national level in order to avoid disparities in access between regions and to pool the financial risk of irregular distribution of patients b) Regional and local funding bodies should liaise and cooperate with national authorities to avoid inconsistencies and inequalities in regional access c) It is preferable that funding for OMPs should come out of normal healthcare budgets rather than from ear-marked rare disease funds that do not allow for a long-term perspective	

Funding for OMPs should be co-ordinated at a national level in order to avoid disparities in access between regions and to pool the financial risk of irregular distribution of patients across geographies. Where reimbursement decisions have been left to regional bodies, variance in access has been observed (e.g. Sweden, Italy) [8, 88, 89]. Due to the genetic nature of many rare diseases, the burden of payment may be concentrated in one area; a national risk-sharing scheme would overcome regional disparities in disease presentation.

In order to facilitate this, regional and local funding bodies should liaise and cooperate with national authorities to avoid inconsistencies and inequalities in regional access. Regional funding bodies should be represented within national bodies, ensuring funding decisions taken at this level are quickly implemented locally.

It is preferable that funding for OMPs should come out of normal healthcare budgets as opposed to ear-marked rare disease funds that do not allow for a long-term perspective. Ear-marked disease-specific funds are seen in countries such as Scotland [90], Italy [91] or in England with the Cancer Drug Fund [92]. These tend to be subject to political revision and can be arbitrary in value.

**Table:** 

**PRINCIPLE 8: Evidence-based funding mechanisms should be developed to guarantee long-term sustainability**	
a) Manufacturers, payers and HTA agencies should collaborate nationally to improve forecasting and cooperate at the European level for horizon scanning with the aim of helping budget holders predict and plan for expenditure and ensure adequate funding of OMPs b) Early stage dialogue should occur between all stakeholders to ensure long term sustainability of outcomes	

Manufacturers, payers and HTA agencies should collaborate nationally to improve forecasting and cooperate at the European level for horizon scanning. Such collaboration should aim to help budget holders predict and plan for expenditure and ensure adequate funding of OMPs. One of the primary concerns of payers regarding OMPs is the long-term sustainability of funding [5, 17]. To ensure funding is sustainable, payers need to model beyond annual budget cycles to forecast the total expenditure on OMPs, including likely savings as older products lose marketing exclusivity. In addition to budget impact forecasting, payers should look beyond the total expenditure on an OMP to include cost offsets from reduced medical resource use resulting from the introduction of the OMP. Manufacturers should collaborate with payers in this process, providing timely information on products under development, label expansions and expected launch dates, as well as aiding in the estimation of potential cost-offsets. Horizon scanning efforts can be co-ordinated at a European level, as the core information on the number of products likely to be approved is common across European countries.

## Principles on OMP European co-ordination

**Table:** 

**PRINCIPLE 9:** **In the future there should be greater co-ordination of OMP value assessment processes at a European level**	
a) While recognising that the reality today is one of national level competence, there is potential for a greater role for co-ordination of certain elements of value assessment in the future at European level b) Rationales for collaboration between European Member States on value assessment could include: • Guarantee more consistency between Member States in the definition and assessment of clinical value • Greater concentration of clinical expertise • Pooling of data on epidemiology • Opportunities for more systematic collection and assessment of data • Reduced duplication of effort at the national level in the re-assessment of value and as such, faster access to medicines for patients c) In order to co-ordinate efforts, Member States should increasingly collaborate and share their knowledge in preparation for local evidence appraisals d) A co-ordinated mechanism should be put in place at the European level to help reduce evidential uncertainties around OMPs and enable rapid and continuous data collection post launch	

While recognising that the reality today is one of national level competence, there is potential for a greater level of co-ordination at the European level with regards to certain elements of value assessment. Rationales for collaboration between Member States on value assessment could include guarantees about greater consistency in the definition and assessment of clinical value, greater concentration of clinical expertise, pooling of data on epidemiology, opportunities for more systematic collection and assessment of data, and reduced duplication of effort at the national level in the re-assessment of value and as such, faster access to medicines for patients. The benefits of establishing European Reference networks for rare diseases has been widely recognised [93], notably by overcoming the limited experience of professionals confronted with rare disease and by improving access for EU citizens to treatment requiring a particular concentration of resources or expertise. The future European collaboration on Relative Effectiveness Assessments, which is currently launching pilots within EUNetHTA’s Joint Action 3 [94] and planning to be formalised in 2019, is a key step forward towards this needed collaboration.

## Conclusion

The advent of European OMP legislation and subsequent growth in new authorised treatments represents an important opportunity to improve human health and redress the years of under-investment in rare disease treatments. To be successful, this goal requires the maintenance of incentives that have stimulated investment in European rare disease research with subsequent benefits for the wider scientific economy [95]. It also requires further development of P&R processes to improve patient access to new medicines and provide greater clarity to those who would invest in future research in rare diseases.

There are many challenges to quick and comprehensive patient access to OMPs [5, 47]. In addition to the technical issues of imperfect information, evidential and financial uncertainty, evolving regulatory pathways and novel scientific platforms, are the more general concerns of lack of mutual understanding between payers and OMP manufacturers. Greater consistency of processes between Member States, clear criteria for the assessment of value and value for money, increased transparency and clarity about decisions, and more collaboration and information sharing between payers and manufacturers could help address these challenges.

The principles outlined in this paper may be helpful in drawing together an emerging consensus on this topic and identifying areas where consistency in payer approach could be achievable and beneficial. These principles recognise that P&R is a national competency, as there needs to be flexibility for payers to reflect local societal preferences. They do not seek to specify the process that payers use to make P&R decisions, but to ensure a common underlying approach and convergence around core concepts. Future research might seek to explore on a country-by-country basis the areas in which existing value assessment and P&R frameworks align with these principles and where there is divergence, as a stimulant for policy appraisal. Identifying differences between the principles and current systems will stimulate further discussions on the practical changes and ways to implement the principles.

The formulation of the principles has been informed by evidence wherever possible, but ultimately these recommendations represent the opinion and experiences of those experts involved in the development, regulation, assessment and use of OMPS in Europe. As such, they are fundamentally subjective. These principles are likely to stimulate the current debate regarding cooperation between HTA agencies in Europe, and to inspire future research to better document what can be achieved to improve a fair assessment of OMPs and a fair utilisation of this assessment by the decision-makers.

The need for greater consistency, clarity and certainty will become ever more important as the number of OMPs grows. Yet, while there are still many thousands of rare diseases for which no effective treatments exist, all stakeholders – payers, policy makers and manufacturers – will need to take a long-term perspective and build frameworks and processes that are capable of addressing the scale of the challenge. Ultimately, OMPs offer the potential to improve equity of access to effective treatment for the biologically least advantaged in our communities. All stakeholders have an obligation to work together to ensure that this promise is realised.

## Additional file

Additional file 1: Glossary of terms of the guide to core elements of value presented in Fig. 3. (DOCX 121 kb)

## Abbreviations

AEG: Additional evidence generation
ASCO: American society of clinical oncology
COMP: Committee for orphan medicinal products
EMA: European medicines agency
EPAR: European public assessment report
ESMO: European society for medical oncology
EU: European Union
EUnetHTA: European network on health technology assessment
EUPATI: European Patients’ Academy
HCP: Healthcare professional
HRQoL: Health-related quality of life
HTA: Health technology assessment
ICER: Incremental cost-effectiveness ratio
MCDA: Multi-criteria decision analysis
MoCA-OMP: Mechanism of coordinated access to orphan medicinal products
MPS: Mucopolysaccharidosis
NICE: National Institute for Health and Care Excellence
OMP: Orphan medicinal product
P&R: Pricing and reimbursement
PACE: Patient and clinician engagement
QALY: Quality-adjusted life year
R&D: Research and development
TC: Teleconference
TVF: Transparent value framework
