# Supplementary material for: Recommendations from the European Working Group for Value Assessment and Funding Processes in Rare Diseases (ORPH-VAL)
Source: Orphanet J Rare Dis. 2017 Mar 10;12:50. doi: 10.1186/s13023-017-0601-9 (PMC5345269; doi:10.1186/s13023-017-0601-9)
Supplement: Additional file 1: — Glossary of terms of the guide to core elements of value presented in Fig. 3. (DOCX 121 kb) [file 13023_2017_601_MOESM1_ESM.docx]

| **Patient level** | Considers elements that are relevant to the patient with the disease only. |
| --- | --- |
| **Existing treatment options** | Current treatment options available, even if this is just supportive care. |
| **Impact of treatment on patient level elements** | The effect of treatment is considered across the four sub-elements listed: survival, morbidity, patient experience and HRQoL, and patient economic burden. The magnitude of therapeutic benefit should be considered, both as an average benefit across the population (mean improvement) and also in terms of the distribution of benefit (proportion of patients who benefit, i.e. responder rate). |
| **Morbidity** | What are the non-life threatening symptoms and manifestations of the disease on the patient? Is it debilitating? What are the severity and frequency of symptoms? Is it a chronic or acute disease? |
| **Patient economic burden** | This is the economic burden that arises from the disease impact on the patient, such as out of pocket medical costs, lost worktime (i.e. lost income) and home adaptation. |
| **Patient experience and HRQoL impairment** | Does the disease reduce the HRQoL of patients, including domains commonly captured in generic and disease-specific HRQoL questionnaires, such as function, daily activities, pain, emotional impact, social impact, patient suffering, etc. |
| **Side effects** | The incidence and severity of side effects from treatment and the impact on patient adherence. Can work positively (less side effects) or negatively (more side effects). |
| **Survival / life expectancy** | Is the disease life threatening? How much does the disease affect a patient’s life expectancy? |
| **Treatment convenience** | The mode, route, formulation and location of administration, and the impact on the patient’s life and their adherence. Can work either positively or negatively. |
| **Healthcare system level** | Includes impact on the whole health system, including primary and secondary care organisations, doctors, pharmacists, nurses, as well as social care (even when this does not fall within the healthcare budget). |
| **Impact of treatment on healthcare system** | The effect of treatment is considered across the two sub-elements mentioned above: healthcare system resource use and healthcare system organisation. |
| **Healthcare system organisation** | The consequences of a disease may have implications for how a healthcare system is structured. For example, if it requires a very specific form of intervention there may be designated facilities for that purpose. |
| **Healthcare system resources and budget** | The impact of the disease on the healthcare system resources and budget, such as the amount of hospital visits, surgeries and diagnostics that patients receive with current standard of care. |
| **Societal level** | This looks at the impact of the disease and treatment beyond the patient and the healthcare system, particularly on carers, family, employers and wider society. |
| **Family/carer economic burden** | Is there any economic impact on carers and family members because of the patient’s disease? Such as out-of-pocket medical costs or lost income. What potential is there for the treatment to alleviate the economic burden of carers? |
| **Family/carer HRQoL** | Is there any impact on HRQoL for carers or family members as a result of the patient’s disease? For example, they may experience an emotional impact, stress or reduced social interaction. What potential is there for the treatment to alleviate the burden on carers and family through improvement in the patient’s health? |
| **Societal economic burden** | There is also a potential economic cost to society due to lower productivity from carers or family members. This economic cost should be considered at an individual level, rather than at an aggregate societal level. |
| **Considerations beyond product value** | In addition to the assessment of the intrinsic value of an OMP, there are other elements that are not linked to the product value, but which are relevant to the pricing and reimbursement decision. |
| **Budget impact** | This is the additional cost (or saving) to the healthcare system as a result of the new therapy, taking into account savings as a result of patient improvement. Budget impact should be considered in both the short and long term and account for future savings after loss of exclusivity/patent. The rarity of the condition has a direct impact on the budget impact of the treatment. |
| **Rarity** | The prevalence of the disease is relevant to the pricing and reimbursement decision due to its impact on the economics of rare disease innovation, the budget impact of the product, and societal preferences. |
| **Societal preferences** | Each country has its own values and social preferences which need to be incorporated into P&R decisions. For example, ideas concerning social solidarity are likely to be relevant to decisions on rare disease treatments. Priorities may be given to certain conditions (Aagaard & Kristensen, 2014) with certain characteristics based on societal preferences resulting in the implementation of incentives and specific healthcare policies. |
| **Sustainability of innovation in rare diseases** | Failing to consider rarity risks removing incentives for manufacturers to develop treatments for more rare conditions where the commercial opportunity is lower. |
| **Uncertainty of OMP value** | The rarity of the condition has potential implications for the ability to generate evidence around the disease and the benefits of treatment. For all of the product value levels, consideration should be given to the quality of the evidence around the elements and the extent of uncertainty of those estimates. Evidence and uncertainty should be considered in light of the rarity of the disease, the extent of the disease knowledge and the disease area infrastructure. |
